# Undescribed Metabolites from an Actinobacteria *Acrocarpospora punica* and Their Anti-Inflammatory Activity

**DOI:** 10.3390/molecules27227982

**Published:** 2022-11-17

**Authors:** Ming-Der Wu, Ming-Jen Cheng

**Affiliations:** 1 Bioresource Collection and Research Center (BCRC), Food Industry Research and Development Institute (FIRDI), Hsinchu 300, Taiwan; 2 Department of Life Science, Fu Jen Catholic University, New Taipei City 24205, Taiwan

**Keywords:** *Acrocarpospora punica*, Streptosporangiaceae, alkaloids, diterpenoid, steroid, anti-NO activities

## Abstract

In an effort to explore bioactive anti-inflammatory compounds from natural *Actinobacteria* resources from all over Taiwan and various ecological environments, an active strain of *Acrocarpospora punica* was collected at Taitung County in Taiwan, prepared from soil origin. A bioassay-guided fractionation of the BuOH extract of a culture broth of a new strain of the actinomycete *Acrocarpospora punica* led to the isolation of five previously undescribed compounds: acrocarpunicains A–F (**1**–**6**). The structures were elucidated by 1D and 2D Nuclear Magnetic Resonance (NMR) spectroscopy and mass spectrometry. Furthermore, the isolated compounds were subjected to in vitro testing to evaluate their anti-inflammatory activity. Of these isolates, acrocarpunicains A (**1**), B (**2**), C (**3**) and F (**6**) showed NO inhibitory activity with IC_50_ values of 9.36 ± 0.25, 10.11 ± 0.47, 5.15 ± 0.18, and 27.17 ± 1.87 μM, stronger than the positive control, quercetin (IC_50_ = 35.95 ± 2.34 μM). To the best of our knowledge, this is the first report on azaphilone and phenanthrene-type metabolites from the genus *Acrocarpospora*.

## 1. Introduction

Actinobacteria (Actinomycetes) can produce various metabolites, which can be used as antibiotics (streptomycin, cycloheximide), anticancer drugs (bleomycin), dextran synthetase inhibitors, immunomodulators (rapamycin), etc. In addition to occupying a pivotal position in agriculture, they also play an increasingly important role in animal husbandry, the food industry, and in environmental protection. They are also well known as an outstanding and fascinating source of commercially valuable bioactive compounds, particularly the aforementioned antibiotics. Almost a half of the known microbial bioactive secondary metabolites are derived from actinomycetes [1,2,3,4,5,6,7], of which more than 70% were obtained from the genus *Streptomyces*. However, the active ingredients of many new Taiwanese *Actinobacteria* and their mechanisms of actions are still unknown. Thus, it is necessary to study the active compounds of these *Actinobacteria* using scientific methods.

In exploring the actinomycetes via preliminary screening, we recently isolated a novel strain, named AC18001 (04107M-2), from the soil of Taitung County with a unique morphology and possessing anti-inflammatory activities. This strain was determined to be *Acrocarpospora punica* based on their phenotypic and genotypic data.

The genus *Acrocarpospora* was first described by Tamura et al. [8] and composed of the following three species: *A. corrugatum*, *A. macrocephala* and *A. pleiomorpha* [8]. This microorganism was isolated and identified by our research team. On the basis of phenotypic and genotypic data, it is proposed that the strain should be classified as a new species, named *A. punica*. Previous chemical investigations of the genus *Acrocarpospora* have received less attention, and only a few articles have reported on the classification of molecular biology [9,10,11].

**Figure 1:**
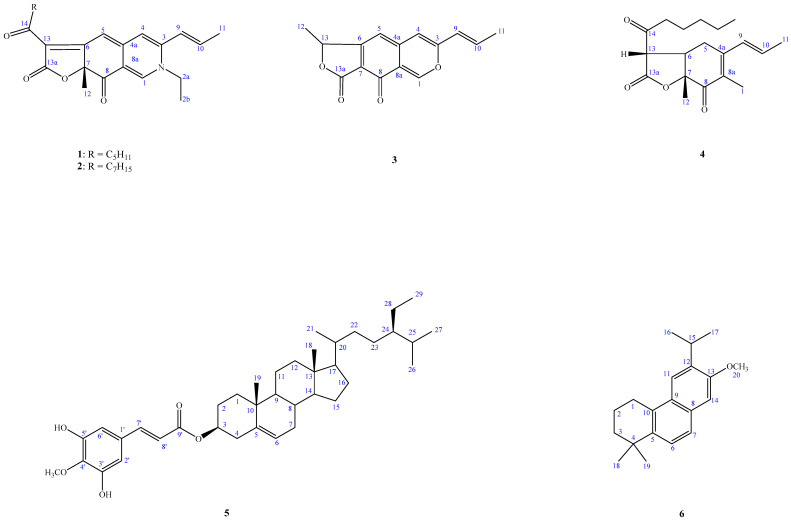
Compounds **1–6**, isolated from *Acrocarpospora punica*.

Recently, over 600 species of microorganisms have been screened for in vitro anti-inflammatory activities, and *A. punica* has been found to be one of the active species. In a continuation of our studies, aimed at finding new chemical and anti-inflammatory constituents from this genus, we firstly describe the isolation and structural elucidation of five previously undescribed compounds: acrocarpunicains A–F (**1**–**6**), from the EtOAc extract of a culture broth of *A. punica* (Figure 1). A bioassay-guided fractionation of the BuOH extract of the whole broth has led to the isolation of A (**1**), B (**2**), C (**3**) and F (**6**) with anti-inflammatory activities, along with the isolation of two inactive ones. We herein report the isolation and anti-inflammatory activity properties of these compounds.

## 2. Results

### 2.1. The Taxonomic Identification (Phenotypic and Genotypic Data) of Acrocarpospora punica

**Figure 2:**
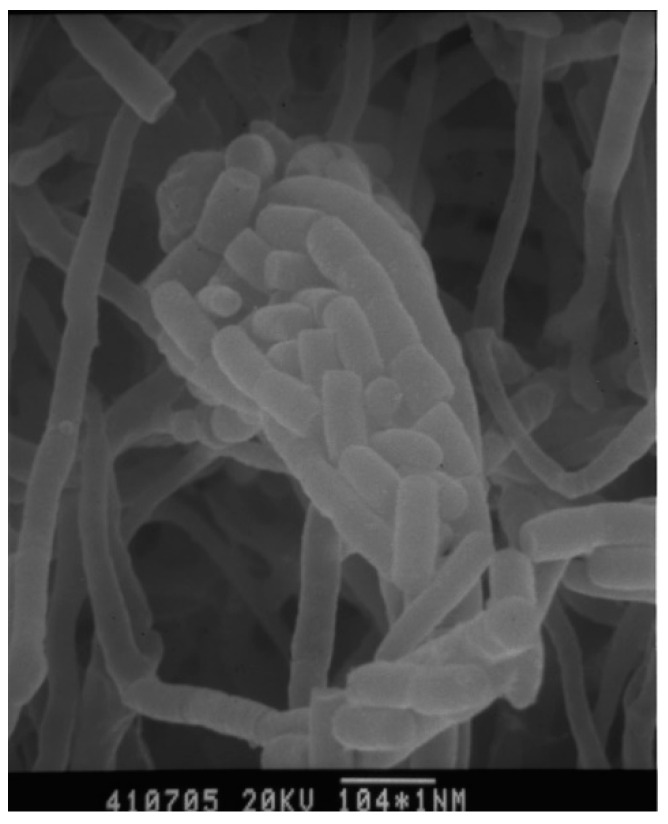
Scanning electron micrographs of strain *A. punica* grown on HV agar for 14 days at 28 °C. Bar 1.5 μm.

Strain 04107M-2^T^ produced branched and non-fragmented substrate mycelia, club-shaped structures borne on the tips of the aerial mycelium. The spores were non-motile, rod and smooth-surfaced (Figure 2). The growths on various media were poor, and a reddish with golden luster crystal had been observed on oatmeal agar. No soluble pigment was produced in all of the media tested. The results of these physiological and biochemical tests are indicated in Table 1.

#### 2.1.1. Cellular Biochemistry

Strain 04107M-2^T^ contained meso-A_2_pm, madurose, arabinose, rhamnose, glucose and ribose in whole-cell hydrolysates. The predominant menaquinone found are MK-9(H_4_), MK-9(H_2_); mycolic acids were not detected. Phosphatidylethanolamine (PE) was detected. The major fatty acid methyl esters were Iso-C_16:0_ (14.82%), C_16:0_ (14.63%), C_17:0_ (13.79%) and 10-methylC_17:0_ (23.77%). The GC content of the DNA was 72.2 mol%.

#### 2.1.2. Phylogeny

**Figure 3:**
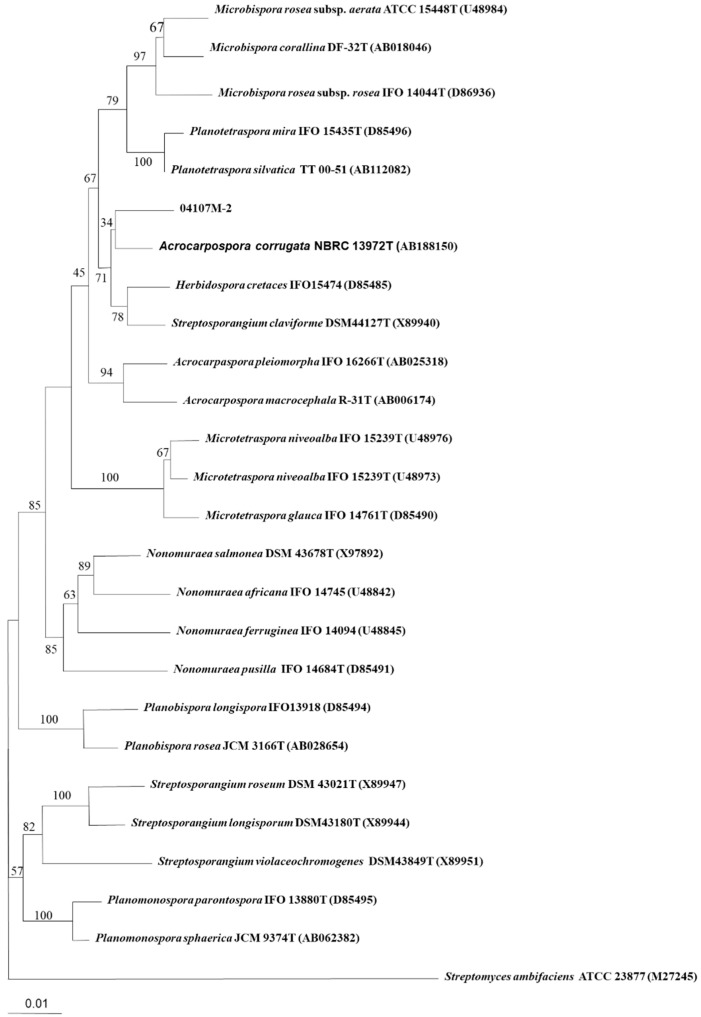
Neighbor-joining tree based on almost complete 16S rDNA sequences showing the phylogenetic position of strain 04107M-2T within the *Acrocarpospora* species. Numbers at nodes indicate percentage of 1000 bootstrap resamplings and only values over 50% are given. Bar, 0.01 substitutions per nucleotide position.

The almost-complete 16S rRNA gene sequence (1511 nt) of strain 04107M-2^T^ was determined. A preliminary comparison of the sequence against the GenBank database revealed high sequence similarity values with members of the genus *Acrocarpospora*. The phylogenetic tree based on the 16S rRNA gene sequences of the strain 04107M-2^T^, other valid published *Acrocarpospora* species, and other related species are shown in Figure 3. The binary similarity values ranged between 96.5% (*A. pleiomorph* IFO 16266^T^) and 98.2% (*A. corrugata* NBRC 13972^T^). The DNA–DNA hybridization rates determined with the new isolate 04107M-2^T^ to its closest type strains of *A. corrugata* BCRC 16357^T^ was 2.9%; *A. macrocephala* was 0.4%, and *A. pleiomorpha* was 1.0% (Table 2). It is clear from the DNA–DNA relatedness (<70%) study that strain BC 44T-5T and these two species belong to separate species [12]. The distinctiveness of the isolate also comes from phenotypic evidence compared with the nearest phylogenetic neighbors (Table 2). On the basis of phenotypic and genotypic characters, it was evident that the isolate should be classified as a new species of the genus *Acrocarpospora*. The name *Acrocarpospora punica* sp. nov., with the type strain 04107M-2^T^ (=BCRC AC 18001^T^).

### 2.2. Structure Elucidation of Compounds

**Figure 4:**
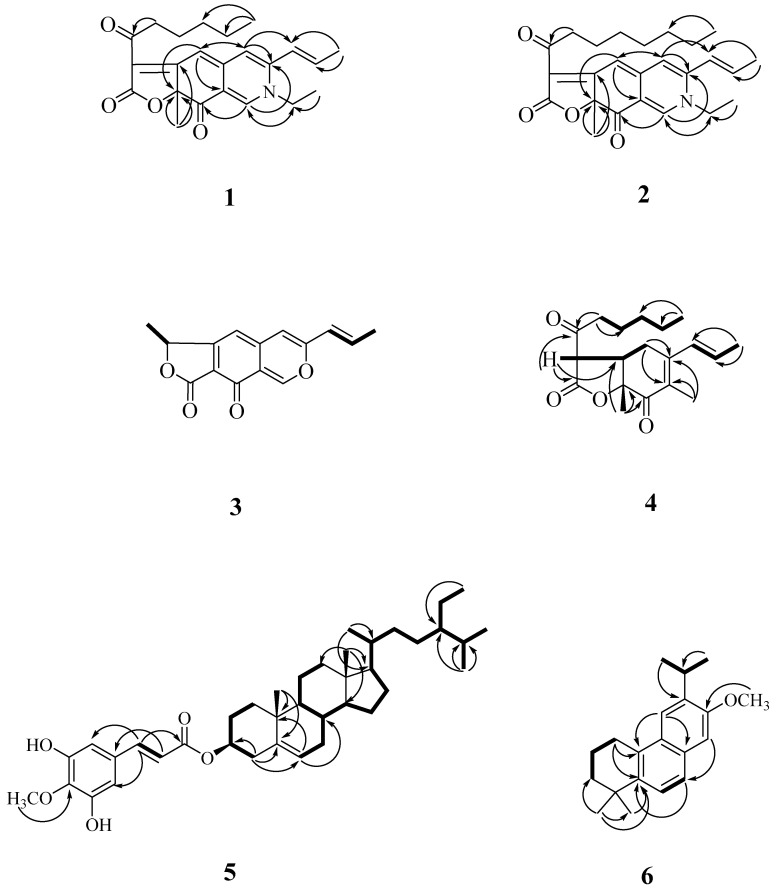
Key COSY (▬) and HMBC (→) correlations of **1**, **2**, and **4**–**6**.

**Figure 5:**
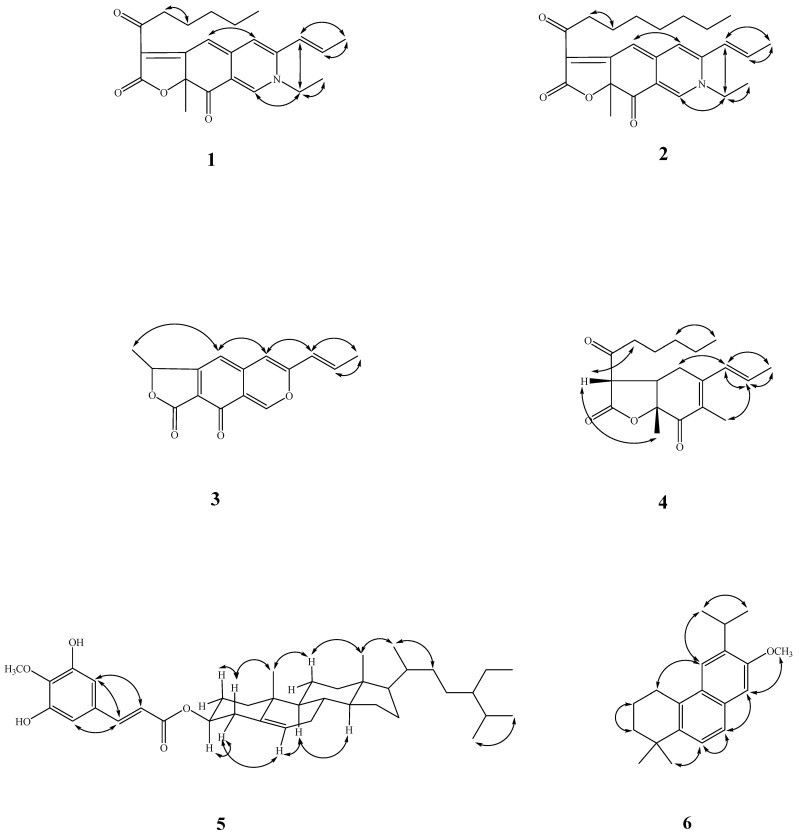
Key NOESY correlations (↔) of **1–6**.

Compound **1** was obtained as optically active oil with [α${]}_{D}^{25}$ = +916 (*c* 0.01, CHCl_3_), and its molecular formula was deduced as C_23_H_27_NO_4_ from HR-ESI-MS data, implying 11 degrees of unsaturation. The UV spectrum depicted the typical absorption of azaphilone bearing a nitrogen atom at λ_max_ (log *ε*) 245, 279, 424, 501 nm [13,14]. Its IR spectrum showed bands corresponding to the absorptions of a conjugated carbonyl group at 1720 and 1622 cm^−1^, respectively. The CD spectrum showed a positive Cotton effect at 217, 283, and 407 nm, and showed a negative Cotton effect at 237, 341, and 534 nm. The ^1^H-NMR spectrum of **1** (Table 3) showed four methyl groups including two primary methyl [δ_H_ 0.86 (3H, t, *J* = 7.2 Hz, H-19), 1.44 (3H, t, *J* = 7.2 Hz, H-2b)], one tertiary methyl [δ_H_ 1.68 (3H, s, H-12)], and one secondary methyl [δ_H_ 2.00 (3H, dd, *J* = 6.6, 1.8 Hz, H-11)], five olefinic protons, including three specific azaphilone olefinic protons [δ_H_ 6.64 (1H, t, *J* = 1.8 Hz, H-4), 6.73 (1H, d, *J* = 1.8 Hz, H-5), 7.77 (1H, d, *J* = 0.6 Hz, H-1)] and a pair of *trans*-olefinic groups [δ_H_ 6.23 (1H, dd, *J* = 15.6, 1.8 Hz, H-9)/6.47 (1H, ddd, *J* = 15.6, 6.6, 1.8 Hz, H-10)], and five methylene groups [1.30 (4H, m, H-17 and H-18), 1.65 (2H, p, *J* = 7.2 Hz, H-16), 2.92 (2H, dq, *J* = 16.2, 7.2 Hz, H-15)]. Comparing the NMR data of **1** with those of rubropunctamine [15] revealed they share a similar structure. The differences between them were that **1** showed another ethyl group [δ_H_ 1.44 (3H, t, *J* = 7.2 Hz, H-2b) and 3.91 (2H, qd, *J* = 7.2, 1.2 Hz, H-2a)] at the N-2 position. This was confirmed by the HMBC correlation from H-2a to C-1. Other COSY, HMBC, and NOESY correlations of **1** (Figure 4 and Figure 5) can confirm the completed structure and named acrocarpunicain A.

Compound **1** was identified by comparison with literature data of monascorubramine but showed a dextrorotatory optical activity with [α${]}_{D}$ = +916 (*c* 0.01, CHCl_3_). Without an enantiomeric antipode for comparison, but with the reference to monascorubramine ((7*R*)-configuration; [α${]}_{D}$ −2600° (c 0.12, CHCl_3_)) [16,17], the stereochemistry at C-7 of **1** would appear to be of the (7*S*)-configuration.

Compound **2** was obtained as reddish oil with an optical rotation +336 (*c* 0.01, CHCl_3_), and the molecular formula was determined as C_25_H_31_NO_4_ based on the HRESIMS peak at *m*/*z* 432.21542 [M+Na]^+^ (calcd. for C_23_H_27_N_1_O_4_Na 432.21508) with 11 degrees of unsaturation. An analysis of the NMR (Table 3), UV, and IR data suggested that the structure of **2** is similar to that of compound **1**. The UV spectrum depicted the typically absorption of red pigments azaphilone bearing a nitrogen atom at λ_max_ (log *ε*) 202 (4.12), 247 (4.00), 286(4.00), 424 (3.96), 503 (4.05) nm [12,13]. The IR spectrum revealed the presence of a conjugated carbonyl group (1724, 1624 cm^−1^). The CD spectrum showed a positive Cotton effect at 220, 285, and 408 nm, and showed a negative Cotton effect at 237, 340, and 547 nm. The ^1^H-NMR spectrum of **2** (Table 3) showed four methyl groups, including one terminal methyl at the side chain group (δ_H_ 0.86), one primary methyl (δ_H_ 1.44), one secondary methyl (δ_H_ 1.68) connected with a pair of *trans*-olefinic groups (δ_H_ 6.23 and 6.47), one tertiary methyl group (δ_H_ 2.00), three specific azaphilone olefinic protons (δ_H_ 6.64, 6.73, and 7.77), and five methylene groups. The NMR data of **2** was similar to those of **1**, except that the integrate of the high-field methylene groups (δ_H_ 1.33) of **2** is two more protons than **1** and **2** depicted an octanoyl side chain group at C-13. Therefore, **2** was supported by the COSY, HMBC, and NOESY correlations showed in Figure 4 and Figure 5. As evidenced by the above data, the entire structure of **2** was confirmed and named acrocarpunicain B.

Isolate **2** showed a dextrorotatory optical activity with +336 (*c* 0.01, CHCl_3_), and by reference to (*R*)-(+)-monascorubramine ([α${]}_{D}$ −2600° (c 0.12, CHCl_3_)), the configuration of **2** at C-7 was proposed as being in the *S*-form.

Compound **3**, isolated as oil with [α${]}_{D}^{25}$: ~0 (*c* 0.01, CHCl_3_), showed a molecular ion [M+H]^+^ peak at *m*/*z* 257.08192 for C_15_H_12_O_4_, corresponding to 10 indices of hydrogen deficiency (IHD). An analysis of its IR spectrum suggested that **3** contained an α,β-unsaturated γ-lactone group (1624 cm^−1^), and conjugated C=O (1742 cm^−1^) moieties. The UV spectrum revealed the absorption band at λ_max_ (log *ε*) 206 (3.80), 232 (3.88), 322 (3.81) nm. The ^1^H-NMR spectroscopic data of **3** (Table 3) exhibited two tertiary methyl groups [*δ*_H_ 1.69 (3H, d, *J* = 7.2 Hz, H-12) and 1.96 (3H, dd, *J* = 6.6, 1.8 Hz, H-11)], one oxymethine [*δ*_H_ 5.62 (1H, q, *J* = 7.2 Hz, H-13)], three singlet olefinic protons [*δ*_H_ 6.30 (1H, s, H-5), 7.36 (1H, s, H-4), and 8.79 (1H, s, H-1)], which were characteristic of an azaphilone skeleton, and a pair of *trans*-olefinic protons [*δ*_H_ 6.10 (1H, dd, *J* = 15.6, 1.8 Hz, H-9) and 6.77 (1H, dd, *J* = 15.6, 6.6 Hz, H-10)]. These UV and ^1^H-NMR spectra suggested **3** is a liner azaphilone skeleton. Comparison of the ^1^H-NMR data of **3** to those of monascorubrin [18] proposed that their structures are closely related, despite the fact that 3-octanoylfuran-2(5*H*)-one of monascorubrin [18] was replaced by 5-methylfuran-2(5*H*)-one in **3**. The NOESY correlation of **3** (Figure 4) can also confirm the structure as (*E*)-1-methyl-7-(prop-1-en-1-yl)-3*H*-furo [3,4-g]isochromene-3,4(1*H*)-dione, and named acrocarpunicain C. Because of the optical inactivity, **3** was also proposed to be racemic.

Compound **4** was isolated as a whitish syrup with an optical rotation +24.1 (*c* 0.075, CHCl_3_). Its HRESIMS showed a molecular formula of C_19_H_26_O_4_, indicating a seven index of hydrogen deficiency (IHD) as determined by an [M+Na]^+^ ion peak at 319.06270 (Calcd.: C_10_H_12_O_4_Na, 319.06278). The IR spectrum exhibited absorption bands at 1786 (α,β-unsaturated γ-lactone group), and 1698 (conjugated ketone group) cm^−1^. The UV spectrum absorption showed λ_max_ (log *ε*) 220 (3.80) nm. The CD spectrum showed a positive Cotton effect at 206 and 224 nm, and a negative Cotton effect at 259 and 308 nm. The ^1^H-NMR spectrum of **4** (Table 3) exhibited the existence of one allylic Me [*δ*_H_ 2.94 (3H, s, H-1)], one singlet methyl [*δ*_H_ 1.47 (3H, s, H-12)], one primary terminal methyl [0.90 (3H, t. *J* = 7.2 Hz, H-19)], one tertiary methyl [*δ*_H_ 1.72 (3H, dd, *J* = 6.6, 1.2 Hz, H-11)] bearing with a pair of *trans*-olefinic groups [*δ*_H_ 5.51 (1H, ddd, *J* = 15.6, 3.0, 1.2 Hz, H-9) and 5.78 (1H, dq, *J* = 15.6, 6.6 Hz, H-10)], two aliphatic CH groups [*δ*_H_ 3.33 (1H, ddd, *J* = 13.2, 11.4, 4.8 Hz, H-6) and 3.73 (1H, d, *J* = 13.2 Hz, H-13)], and four CH_2_ H-atoms of one ketone [*δ*_H_ 1.31 (4H, m, H-17 and H-18), 1.64 (2H, p, *J* = 7.2 Hz, H-16), 2.62 (1H, dt, *J* = 18.0, 7.2 Hz, H-15b) and 3.03 (1H, dt, *J* = 18.0, 7.2 Hz, H-15a)]. The ^13^C-NMR and DEPT spectra displayed 19 carbon resonances, which were four methyl groups [*δ*_C_ 13.9 (C-19), 17.2 (C-12), 17.8 (C-11), and 22.4 (C-1),], five methylenes [*δ*_C_ 22.4 (C-18), 22.5 (C-17), 22.8 (C-16), 38.5 (C-5), and (C-15)], two methines [*δ*_C_ 43.7 (C-6) and 54.4 (C-13)], four olefinic carbons [*δ*_C_ 128.3 (C-9), 130.1 (C-10), 148.9 (C-8a), and 156.1 (C-4a)], and three C=O groups including one saturated ketone group [*δ*_C_ 201.3 (C-14)], one α,β-unsaturated CO group [*δ*_C_ 191.8 (C-8)], and one lactone group at [*δ*_C_ 168.7 (C-13a)]. Compound **4** was speculated to have two rings (a six-membered and a five-membered ring) on account of five out of seven unsaturation equivalents were accounted for by the abovementioned ^13^C-NMR. The ^1^H-NMR and ^13^C-NMR spectra of compound **4** showed similar features to those of monascuspurpurone [19], and allowed the skeleton of **4** to be 5,13-dimethyl-3a,7a-dihydro-3*H*,4*H*-benzofuran-2,4-dione. Based on the COSY correlation (Figure 2) from H-9 (*δ*_H_ 5.51)—H-10 (*δ*_H_ 5.78)—H-11 (*δ*_H_ 1.72), H-5 (*δ*_H_ 2.32/2.92)—H-6 (*δ*_H_ 3.33)—H-13 (*δ*_H_ 3.73), and H-15 (*δ*_H_ 2.62/3.03) to H-19 (*δ*_H_ 0.90), the assignment of these three fragments were confirmed. The HMBC correlation (Figure 2) of H-1 to C-4a and C-8a; and H-12 to C-7 and C-8, these two singlet methyl groups were assigned to C-8a and C-7, respectively. The side chain group from C-15 to C-19 used C-14 to connect with the furanone ring based on the HMBC correlation from H-15 to C-14 and from H-13 to C-14. The 2-oxopentyl moiety at C-4a in monascuspurpurone [19] was replaced by *trans*-propenyl in **4**, certainly. The *trans*-fused ring linkage of these two rings was revealed by the absence of a NOESY correlation between H-12 and H-6. Moreover, the H-12 showed a NOESY correlation to H-13, referring to their suprafacial orientation. As a result, the relative configuration of **4** was assigned as *rel*-(6*R*, 7*R*, 13*S*) [19]. Based on these results, **4** was identified and named acrocarpunicain D.

Compound **5** was obtained as colorless needles. It has a molecular formula C_39_H_58_O_5_ as determined by the HREIMS (*m*/*z* 606.4286 [M]^+^, calcd. for C_39_H_58_O_5_, 606.4286), corresponding to 11 indices of hydrogen deficiency. Its UV–VIS spectrum has maximum absorption bands at 326 nm (log ε = 3.92) and 227 nm (log ε = 3.89). The IR spectrum exhibited the absorption bands of hydroxy (3418 cm^−1^), and conjugated ester, C=O (1720 cm^−1^) functionalities. 

In the ^1^H-NMR spectrum of **5**, typical signals assignable for two quaternary carbons [δ_H_ 1.04 (3H, s, Me-19), 0.68 (3H, s, Me-18)], three tertiary carbons [δ_H_ 0.92 (3H, d, *J* = 6.0 Hz, Me-21), 0.84 (3H, d, *J* = 6.8 Hz, Me-27), 0.81 (3H, d, *J* = 6.8 Hz, Me-26)], one secondary [δ_H_ 0.82 (3H, d, *J* = 8.0 Hz, Me-29)], one tertiary carbon connected to an oxygen atom [δ_H_ 4.72 (1H, m, H-3)], a trisubstituted double bond resonance [δ_H_ 5.39 (1H, br s, H-6)], two *meta* hydrogens on the benzene ring [δ_H_ 6.80 (1H, d, *J* = 2.0 Hz, H-2), 6.64 (1H, d, *J* = 2.0 Hz, H-4)], and an (*E*)-double bond conjugated with an ester group [δ_H_ 7.49 (1H, d, *J* = 16.0 Hz, H-7), 6.24 (1H, d, *J* = 16.0 Hz, H-8)]. In its ^13^C-NMR spectrum (Table 4), 39 resonances were subclassified by DEPT experiments into 7 methyls, 11 methylenes, 13 methines (1 oxygenated methine and 5 sp^2^ methines), and 8 quaternary carbons (1 ester, 3 oxygenated and 1 sp^2^ carbons). All signals in the above spectra showed that compound **5** had a β-sitosterol skeleton with one *O*-linkage isoferulate group. From the signal of the HMBC spectrum, it can be observed that δ_H_ 7.49 (H-7′) is correlated to δ_C_ 108.7 (C-2′)) and 102.5 (C-6′), and δ_H_ 6.22 (H-8′) is correlated to δ_C_ 166.0 (C-9′) and 126.5 (C-1′). Two hydroxyl groups (δ_H_ 5.40 and 5.98 (each 1H, br s)), and a methoxyl group (δ_H_ 3.92 (3H, s, OMe-4′)) located at the aromatic ring were determined by ^13^C-NMR signals at C-3′ (δ_C_ 143.9), C-4′ (δ_C_ 147.4), and C-5 (δ_C_ 134.0) and the HMBC correlations of OMe–C(4)/C-4. From the above, it can be inferred that **5** is a steroid and takes a *trans*-3-hydroxyisoferulate at the C-3 position. It was compared with β-sitosteryl *trans*-isoferulate, and hence, compound **5** was determined to be β-sitosteryl *trans*-3-hydroxyisoferulate and designated as acrocarpunicain E.

Compound **6** was isoalted as oil with [α${]}_{D}^{25}$ = −44.9 (*c* 0.18, CHCl_3_). The molecular formula was determined as C_20_H_26_O on the basis of the [*M*]^+^ peak at *m*/*z* 282.2044 (calcd. 282.2042 for C_20_H_26_O) in its HR-EI-MS. UV absorptions (λ_max_ bands at 244 nm) confirmed the presence of a benzene nucleus [20]. The IR spectrum revealed the presence of aromatic rings (1626, 1483 cm^−1^). Eight indices of hydrogen deficiency (IHD) were determined from the molecular formula, ^13^C-NMR (Table 3), and DEPT spectra. The ^1^H and ^13^C-NMR spectra of **6** showed the typical signals for a phenanthrene: two *para*-hydrogens on the benzene ring [δ_H_ 7.16 (3H, s, H-11), 7.53 (3H, s, H-14), δ_C_ 101.3 (C-11), 125.3 (C-14)], one aromatic OMe [δ_H_ 3.96 (3H, s, OCH_3_-13), δ_C_ 55.2 (OCH_3_-13)], one iPr group [δ_H_ 3.38 (1H, sep, *J* = 6.8 Hz, H-15), 1.18 (3H, d, *J* = 6.8 Hz, H-16), 1.18 (3H, d, *J* = 6.8 Hz, H-17), δ_C_ 27.0 (C-15), 22.8 (C-16 and 17)], two singlet methyl signals [δ_H_ 1.33 (6H, s, CH_3_-18/19), δ_C_ 31.5 (C-18/19)] two mutually coupled aromatic ring protons [δ_H_ 7.32 (1H, d, *J* = 8.2 Hz, H-6), 7.55 (3H, d, *J* = 8.2 Hz, H-7), δ_C_ 122.7 (C-6), 125.0 (C-7)], six quaternary carbons (δ_C_ 34.2 (C-4), 142.2 (C-5), 126.8 (C-8), 131.6 (C-9), 129.0 (C-10), 137.4 (C-12)), and one quaternary oxygenated carbon (δ_C_ 156.2). The three aliphatic CH_2_ at δ_H_ 3.01 (2H, t, *J* = 6.4 Hz, H-1), 1.93 (2H, m, H-2), and 1.70 (2H, m, H-3) were determined by the two-dimensional spectra HMBC and COSY, respectively. Thus, the structure of **6** was determined to be 6-isopropyl-7-methoxy-1,1-dimethyl-1,2,3,4-tetrahydrophenanthrene and was designated as acrocarpunicain F. 

## 3. Discussion

In summary, actinomycetes have been recognized as a large microbial reservoir that can be expected to provide a variety of structurally unique and biologically potent natural metabolites. Continuing our previous chemical and biological studies of microbially produced metabolites, a new actinomycete strain *Acrocarpospora punica,* isolated from soil samples collected in Taitung County, Taiwan was determined to be able to produce bioactive metabolites during its liquid fermentation process according to our system screening plan.

Secondary metabolites of the genus *Acrocarpospora* have been rarely studied in the past. The *A*. *punica* strain in this study has only 10 components reported by our team in the past [21]. After modifying the fermentation conditions, we obtained six components from the active layer of BuOH, five of which were new compounds whose backbones included azaphilone, dihydrobenzofuran, isochromene-3,4(1*H*)-dione, and tetrahydrophenanthrene. These components were originally discovered from the chemotaxonomically significant genus *Acrocarpospora*. These results demonstrate that *Acrocarpospora* produces unique and diverse metabolites in different fermentation conditions and soil-derived collections. Therefore, in a special ecological environment, more natural products with biological activity may be found by searching for *Acrocarpospora* species.

### Biological Studies

**Figure 6:**
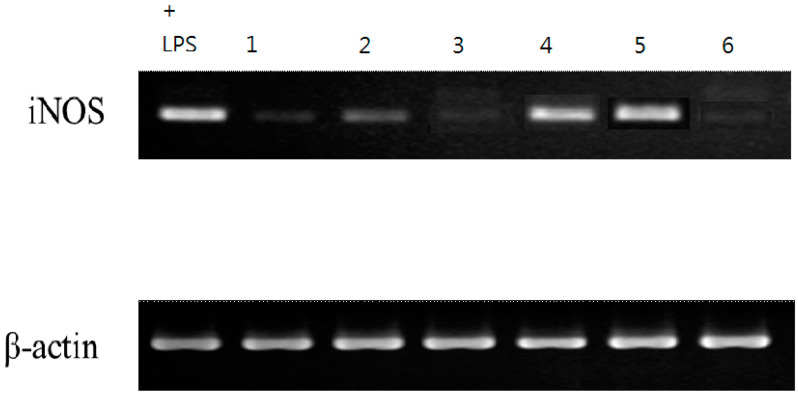
Samples **1**, **2**, **3**, and **6** in the RT-PCR experiment. It can also be clearly seen that the iNOS gene has been significantly inhibited.

The six isolates, in sufficient amounts, were evaluated by examining their inhibitory effects on LPS-induced inducible nitric oxide synthase (iNOS)-dependent NO production in the murine macrophage cell line RAW 264.7 (Table 5). The inhibitory activity data of the six isolated compounds on NO generation by macrophages are shown in Table 3. From the results of our abovementioned tests, the following conclusions can be drawn: (*a*) Compared to quercetin (IC_50_ value 35.95 ± 2.34 μM), which was used as a positive control in this study, acrocarpunicains A, B, C, and F (**1**, **2**, **3** and **6**) exhibited NO inhibitory activity with IC_50_ values of 9.36 ± 0.25, 10.11 ± 0.47, 5.15 ± 0.18, and 27.17 ± 1.87, respectively. (*b*) Compounds **1**, **2**, **3**, and **6** showed about 4-, 3-, 7- and 1.2-fold NO inhibitory activities compared to quercetin, respectively. (*c*) Compound **4** showed weak NO inhibitory activity, whereas compound **5** displayed no NO inhibitory activity. (*d*) Compound **1** (acrocarpunicain A) with a hexanoyl side chain exhibited more effective inhibition than its analogue, compound **2** (acrocarpunicain B), with an octanoyl side chain against LPS-induced NO generation. (*e*) Compounds **3** and **6** belong to liner azaphilone skeleton and naphthenic derivatives, respectively; compound **3** has better anti-inflammatory activity than **6.** (*f*) Furthermore, the RT-PCR analysis in the present study indicated that LPS treatment increased the level of iNOS mRNA expression. Samples **1**, **2**, **3**, and **6** can inhibit the production of NO in the ELISA experiment (Table 5), and in the RT-PCR experiment, the iNOS gene has a significantly inhibited band (Figure 6), the results show that transcription can be inhibited at the same time. In addition, in its effect on translation, it can be seen that the expression of the protein is inhibited by inhibiting the expression of the gene, indicating that samples **1**, **2**, **3**, and **6** may have the effect of regulating immunity. Further, the iNOS result was proven by RT- PCR, demonstrating that the compounds can inhibit the expression of the iNOS gene, and it was noted that these compounds (**1**, **2**, **3**, and **6**) inhibited this increase in a concentration-dependent manner. At the highest concentration, none of the compounds tested showed any obvious cytotoxicity toward RAW 264.7 cells. (*g*) The cytotoxic effects were measured using an MTT assay. The high cell viability (> 90%) indicated that the inhibitory activities of LPS-induced NO production by the active compounds (**1**, **2**, **3**, and **6**) were not as a result of cytotoxicity.

## 4. Materials and Methods

### 4.1. General Experimental Procedures

For the TLC, we used silica gel *60 F_254_* precoated plates (Merck); for column chromatography (CC), silica gel *60* (70–230 or 230–400 mesh, Merck) and Spherical C18 100A Reversed Phase Silica Gel (RP-18) (particle size: 20–40 μm) (Silicycle). For the HPLC, we used a spherical C18 column (250 × 10 mm, 5μm) (Waters) and LDC-Analytical-III apparatus. For the UV spectra, we used a Jasco UV-240 spectrophotometer, λ_max_ (log ε) in nm. For the optical rotation, we used a Jasco DIP-370 polarimeter, in CHCl_3_. For the IR spectra, we used a Perkin-Elmer-2000 FT-IR spectrophotometer; ν in cm^−1^. For the ^1^H-, ^13^C- and 2D-NMR spectra, we used Varian-Mercury-500 and Varian-Unity-Plus-400 spectrometers; *δ* in ppm rel. to Me_4_Si, *J* in Hz. For the ESI and HRESIMS, we used a Bruker APEX-II mass spectrometer, in *m*/*z*.

### 4.2. Microorganism, Cultivation, and Preparation of the Actinobacteria Strain

The strain was isolated from a soil sample collected in Taitung County, Taiwan, by using HV agar, and was then incubated at 28 °C for 3 weeks. The strain was maintained on oatmeal agar and a suspension of spores or mycelia fragments of the strain in a broth containing 20% (*v*/*v*) glycerol was stored at −20 °C. The inoculum’s medium contained: malt extract, 3 g; yeast extract, 3 g; glucose, 5 g; agar, 1.5 g; and distilled water 1 L. The initial pH of the medium was 8. The synthetic culture medium contained: glucose, 20 g; Monosodium Glutamate (MSG), 10 g; K_2_HPO_4_, 5 g; KH_2_PO_4_, 5 g; MgSO_4_•7H_2_O, 1.0 g; KCl, 0.5 g; ZnSO_4_•7H_2_O, 0.01 g; FeSO_4_•7H_2_O, 0.01g; MnSO_4_•H_2_O, 0.003 g per liter of distilled water. The initial pH of the medium was adjusted to 5.5. The slant culture was kept on PDA (potato dextrose agar) Difco. The spores of the strains were prepared by growth on PDA slants for 14 days at 28 °C. The spores were washed with sterile water. A suspension of 10^7^ spores was used to incubate a 5 L Erlenmeyer flask containing 2 L inoculum medium, which was incubated at 28 °C on a rotary shaker for 3 days. This inoculum was then transferred to a 50 L fermenter (B. Braun, Germany) containing 30 L of synthetic medium operated at 100 rpm and at 30 °C with an aeration rate of 0.3 vvm. After 21 days of cultivation, the fermentation was stopped, and the liquid culture was separated from the mycelium by filtration.

### 4.3. Isolation and Characterization of Secondary Metabolites

The culture filtrate (5 l) was repeatedly extracted with BuOH three times. Evaporation of the solvent afforded a dark brown crude extract (30.4 g), which was chromatographed on silica gel and eluted with CH_2_Cl_2_, and the polarity was gradually increased with EtOAc, acetone and MeOH to furnish 10 fractions (1 to 10). Fraction 1 (1622 mg) was purified by MPLC, eluting with CH_2_Cl_2_-EtOAc (8:1) to afford acrocarpunicain A (**1**) (3.9 mg). Fraction 2 (2.8 g) was subjected to Sephadex LH-20 and eluted with MeOH/H_2_O (1:1) to give 7 fractions (2-1 to 2-7). Fraction 2-3 (32.4 mg) was purified by MPLC to produce acrocarpunicain B (**2**) (3.1 mg). Fraction 4 (158 mg) was subjected to Sephadex LH-20 and eluted with MeOH to give acrocarpunicain D (**4**) (3.8 mg). Fraction 8 (4.09 g) was subjected to silica gel and eluted with CH_2_Cl_2_, and then enriched with EtOAc to give 5 fractions (8-1–8-5). Fraction 8-3 (814 mg) was chromatographed on a CC (30 g, SiO_2_, 230–400 mesh; *n*-hexane/acetone 2:1) to afford acrocarpunicain E (**5**) (3.6 mg). Fraction 9 (2.69 g) was subjected to silica gel chromatography and eluted with CH_2_Cl_2_-MeOH step gradients to give acrocarpunicain C (**3**) (4.8 mg) and acrocarpunicain F (**6**) (2.7 mg).

Acrocarpunicain A (**1**): Red oil; [α${]}_{D}^{25}$= +916 (*c* 0.01, CHCl_3_); UV (MeOH): λ_max_ (log *ε*) = 245 (4.03), 279 (3.97), 424 (3.85), 501 (3.94) nm; IR (Neat): *v*_max_ = 1720, 1622 (C=O) cm^−1^; ^1^H-NMR: See Table 3. ^13^C-NMR (CDCl_3_, 150 MHz): ^13^C-NMR (150 MHz, CDCl_3_): see Table 4. HRESI-MS *m*/*z*: 404.18399 [M+Na]^+^, C_23_H_27_NO_4_Na, Calcd.: C_23_H_27_N_1_O_4_Na, 404.18378.

Acrocarpunicain B (**2**): Red oil. [α${]}_{D}^{25}$: +336 (*c* 0.01, CHCl_3_). UV (MeOH) λ_max_ (log *ε*) 202 (4.12), 247 (4.00), 286(4.00), 424 (3.96), 503 (4.05) nm. IR *v*_max_ (Neat) 1724, 1624 (C=O) cm^−1^; ^1^H-NMR: See Table 3. ^13^C-NMR (CDCl_3_, 150 MHz): ^13^C-NMR (150 MHz, CDCl_3_): See Table 4. HRESI-MS *m*/*z*: 432.21542 [M+Na]^+^, C_25_H_31_N_1_O_4_Na, Calcd.: C_23_H_27_NO_4_Na, 432.21508.

Acrocarpunicain C (**3**): Oil; [α${]}_{D}^{25}$: ~0 (*c* 0.01, CHCl_3_); UV (MeOH) λ_max_ (log *ε*) 206 (3.80), 232 (3.88), 322 (3.81) nm; IR *v*_max_ (Neat) 1742, 1624 (C=O) cm^−1^; ^1^H-NMR (600 MHz, CDCl_3_): See Table 3; ^13^C-NMR (150 MHz, CDCl_3_): See Table 4; HRESI-MS *m*/*z*: 257.08192 [M+H]^+^, C_15_H_13_O_4_, Calcd.: C_15_H_13_O, 257.08138.

Acrocarpunicain D (**4**): Whitish syrup. [α${]}_{D}^{25}$: +24.1 (*c* 0.075, CHCl_3_). UV (MeOH) λ_max_ (log *ε*) 200 (3.86), 220 (3.80) nm. IR *v*_max_ (Neat) 1786, 1698 (C=O) cm^−1^ CD (MeOH) λext (Δε) 206 (Δε + 5.64), 224 (Δε + 3.33), 259 (Δε − 1.89), 308 (Δε − 1.25) nm. ^1^H-NMR (600 MHz, CDCl_3_): See Table 3; ^13^C-NMR (150 MHz, CDCl_3_): See Table 4. HRESI-MS *m*/*z*: 319.06270 [M+Na]^+^, C_19_H_26_O_4_Na, Calcd.: C_10_H_12_O_4_Na, 319.06278.

Acrocarpunicain E (**5**): Colorless needles; mp.: 100−103 °C; [α${]}_{D}^{25}$ = +15.9 (*c* 0.01, CHCl_3_); UV (MeOH): 227 (3.89), 326 (3.92) nm; IR (KBr): 3418 (OH), 1720 (conjugated ester C=O) cm^−1^; ^1^H-NMR (600 MHz, CDCl_3_): See Table 3; ^13^C-NMR (150 MHz, CDCl_3_): See Table 4); EIMS *m*/*z* (%): 607 ([M+1]^+^, 4), 550 (3), 397 (100), 255 (20), 193 (85), 136 (80), 105 (80); HREIMS *m*/*z* 606.4286 [M−H]^+^ (calcd. for C_39_H_58_O_5_, 606.4286).

Acrocarpunicain F (**6**): oil; [α${]}_{D}^{25}$ = −44.9 (*c* 0.18, CHCl_3_); IR (Neat): 1630, 1519, 1483 (aromatic ring) cm^−1^; ^1^H-NMR (600 MHz, CDCl_3_): See Table 3; ^13^C-NMR (150 MHz, CDCl_3_): See Table 4); EIMS (70 eV) *m*/*z* (%): 282 ([M]^+^, 85), 267 (25), 213 (18), 154 (100), 136 (62), 107 (21); HREIMS *m*/*z* 282.2044 [M+H]^+^ (calcd. for C_20_H_26_O, 282.2042).

### 4.4. Determination of NO Production and Cell Viability Assay

Mouse macrophage cell line (RAW 264.7) was obtained from the Bioresource Collection and Research Center (BCRC 60001) and cultured at 37 °C in Dulbecco’s Modified Eagle’s Medium (DMEM) supplemented with 10% fetal bovine serum (Gibco), 4.5 g/L glucose, 4 mM glutamine, penicillin (100 units/mL), and streptomycin (100 μg/mL) in a humidified atmosphere in a 5% CO_2_ incubator. The cells were treated with 10, 25, 50 μM natural products in the presence of 1 μg/mL LPS (lipopolysaccharide, Sigma-Aldrich) for 20 h. The concentration of NO in culture supernatants was determined as nitrite, a major stable product of NO, by a Griess reagent assay [22], and the cell viabilities were determined using the MTT assay as described previously [23].

### 4.5. Reverse Transcription-PCR

The ImProm-II Reverse Transcription System (Promega) was used to take 1 μg of the extracted total RNA, to which was added 1 μL of Oligo(dT)15 Primer, and other supplements with DEPS water. At this time, the total volume was 5 μL. The reaction was immediately placed on ice for at least 5 min, and then 5 × buffer 4 μL, MgCl2 (25 mM) 1.2 μL, dNTP Mix (10 mM) 1 μL, RNasin Ribonuclease Inhibitor 0.5 μL, ImProm-II reverse transcriptase 1 μL and DEPC H2O 6.8 μL was added; the cDNA product was obtained after the reactions of 25 °C, 5 min; 42 °C, 1 h; and 70 °C, 15 min. At the same time, a negative control group was made without reverse transcriptase to confirm whether there was DNA contamination. The obtained cDNA product was then amplified by a PCR reaction to amplify the predicted fragment.

### 4.6. Real-Time PCR

Using the ABI PRISM 7700 Sequence Detection System, the cDNA product was assayed for iNOS, TNF-α, IL-6 and β-actin (internal standard) in a total reaction volume of 20 μL, including 2 × IQ2 SYBR Green Fast qPCR Master Mix (2X) 10 μL, 10 μM of 5′ and 3′ primers 1 μL each. The cDNA template 1 μL reaction conditions were 95 °C, 2 min, 1 cycle; 95 °C, 5 s; and 60 °C, 30 s, 40 cycles. The primers of the PCR reaction were: sense strand iNOS, 5′-GGA GTG ACG GCA AAC ATG ACT-3′; anti-sense strand iNOS, 5′-TCG ATG CAC AAC TGG GTG AAC-3′; sense strand IL-6, 5′-CAG AGT CCT TCA GAG AGA TAC AG-3′; anti-sense strand IL-6, 5′-GAT GAA TTG GAT GGT CTT GGT CC-3′; sense strand TNF-α, 5′-CAG ACC CTC ACA CTC AGA TC-3′; anti-sense strand TNF-α, 5′-CTT TGA GAT CCA TGC CGT TG-3′; sense strand β-actin, 5′-GGC TGT ATT CCC CTC CAT CG-3′; and anti-sense strand β-actin, 5′-CCA GTT GGT AAC AAT GCC ATG T-3′.

## 5. Conclusions

Actinomycetes have potential economic and biotechnological value and have long been recognized as major microorganisms in the medical industry. To date, there are tens of thousands of antibiotics produced by microorganisms, of which more than 70% are derived from *Actinobacteria* [23]. Secondary metabolites of *Actinobacteria* have various structures and biological activities, including antibacterial, antifungal, antitumor, insecticidal and herbicidal, enzyme inhibition, immune regulation, etc. [24,25], which indicates that *Actinobacteria* have great potential for the development of new medicines. As part of our investigations aimed at exploring structurally novel bioactive secondary metabolites from actinomycetes, our chemical research on the fermentation extract of *Acrocarpospora punica* led to the isolation of five previously undescribed compounds, namely, acrocarpunicain A–F (**1**–**6**) (Figure 2). The structure of these isolates was determined by spectroscopic experiments. The BuOH soluble fraction from the *A*. *punica* fermentation broth was tested in vitro and demonstrated anti-inflammatory activity that decreased LPS-stimulated nitric oxide (NO) in RAW 264.7 cells. In addition, compounds **1**, **2**, **3**, and **6** showed potent inhibition with IC_50_ values ≤27.17 μM, against lipopolysaccharide (LPS)-induced nitric oxide (NO) generation, stronger than the positive control, quercetin (IC_50_ = 35.95 ± 2.34 μM). To the best of our knowledge, this is the first report on azaphilones (**1**–**3**) and phenanthrene analogs (**6**) from the genus *Acrocarpospora*.

## Figures and Tables

**Table 1:** The physiological characteristics of strain 04107M-2^T^.

Characteristics	Reaction *
Growth temperature (°C)	20–30
Decomposition of:	
Adenine	−
Aesculin	+
Casein	+
Hypoxanthine	−
L-tyrosine	−
Xanthine	−
Production of:	
Amylase	−
Melanin	−
Nitrate reductase	−
Urease	+

* +: positive reaction, −: negative reaction.

**Table 2:** DNA hybridization rate among species of *Acrocarpospora*.

Probe	04170M-2^T^ (%)	*A. corrugata*^T^ (%)
04107M-2^T^	100	14.9
*A. corrugata* ^T^	12.8	100.0
*A. macrocephala* ^T^	8.4	4.5
*A. pleiomorpha* ^T^	0.9	7.8

**Table 3:** ^1^H-NMR data for Compounds **1**–**6** in CDCl_3_ (*δ* in ppm, *J* in Hz, 600 MHz in CDCl_3_).

No	1	2	3	4	5	6
	δ_H_	δ_H_	δ_H_	δ_H_	δ_H_	δ_H_
1	7.77, d (0.6)	7.77, d (0.6)	8.79, s	2.94, s	1.84, m 1.12, m	3.01, t (6.4)
2					1.88, m 1.61, m	1.93, m
2a	3.91, qd (7.2, 1.2)	3.91, qd (7.2, 1.2)				
2b	1.44, t (7.2)	1.44, t (7.2)				
3					4.70, m	1.70, m
4	6.64, t (1.8)	6.64, t (1.8)	7.36, s		2.38, m 2.36, m	
5	6.73, d (1.8)	6.73, d (1.8)	6.30, s	2.32, m 2.92, ddd (16.2, 4.8, 2.4)		7.32, d (8.2)
6				3.33, ddd (13.2, 11.4, 4.8)	5.36 (1H, br s)	7.55, d (8.2)
7					1.54 (1H, m) 2.16 (1H, m)	
8					1.48 (m)	
9	6.23, dd (15.6, 1.8)	6.23, dd (15.6, 1.8)	6.10, dd (15.6, 1.8)	5.51, ddd (15.6, 3.0, 1.2)	0.96 (m)	
10	6.47, ddd (15.6, 6.6, 1.8)	6.47, ddd (15.6, 6.6, 1.8)	6.77, dd (15.6, 6.6)	5.78, dq (15.6, 6.6)		
11	2.00, dd (6.6, 1.8)	2.00, dd (6.6, 1.8)	1.96, dd (6.6, 1.8)	1.72, dd (6.6, 1.2)	1.50, m 1.44, m	7.16, s
12	1.68, s	1.68, s	1.69, d (7.2)	1.47, s	2.02, m 1.98, m	
13			5.62, q (7.2)	3.73, d (13.2)		
14					1.02, m	7.53, s
15	2.92, dq (16.2, 7.2)	2.92, dq (16.2, 7.2)		2.62, dt (18.0, 7.2) 3.03, dt (18.0, 7.2)	1.54, m 1.12, m	3.38, sep (6.8)
16	1.65, p. (7.2)	1.65, p. (7.2)		1.64, p. (7.2)	1.84, m 1.16, m	1.18, d (6.8)
17	1.30, m	1.30, m		1.31, m	1.10, m	1.18, d (6.8)
18	1.30, m	1.30, m		1.31, m	0.68, s	1.33, s
19	0.86, t (7.2)	1.30, m		0.90, t (7.2)	1.04, s	1.33, s
20		1.30, m			1.34, m	3.96, s
21		0.86, t (7.2)			0.92, d (6.0)	
22					1.05, m	
23					1.58, m	
24					0.92, m	
25					1.48, m	
26					0.81, d (6.8)	
27					0.84, d (6.8)	
28					1.26, m	
29					0.82, d (8.0)	
1′						
2′					6.80, d (2.0)	
3′						
4′						
5′						
6′					6.64, d (2.0)	
7′					7.49, d (16.0)	
8′					6.22, d (16.0)	

**Table 4:** ^13^C-NMR data for Compounds **1**–**6** (*δ* in ppm, 150 MHz for ^13^C-NMR in CDCl_3_).

No	1	2	3	4	5	6
	δ_C_	δ_C_	δ_C_	δ_C_	δ_C_	δ_C_
1	140.5	140.5	154.9	22.4	37.4	26.6
2					28.7	19.9
2a	49.8	49.8				
2b	15.6	15.6				
3	147.3	147.3	155.8		74.2	38.9
4	117.3	117.3	118.2		38.9	34.2
4a	148.0	148.5	142.5	156.1		
5	97.7	97.7	102.7	38.5	139.8	142.2
6	173.4	173.4	155.8	43.7	122.2	122.6
7	86.0	86.0	121.7	83.4	30.3	125.0
8	194.5	194.4	205.0	191.8	32.9	126.6
8a	117.3	117.3	125.0	148.9		
9	121.4	121.4	129.0	128.3	50.1	131.6
10	138.9	138.9	134.8	130.1	36.7	128.8
11	19.1	19.1	18.5	17.8	21.4	102.9
12	30.1	30.1	20.4	17.2	40.2	137.4
13	105.3	105.9	77.4	54.4	42.5	156.2
13a	170.0	170.0	168.5	168.7		
14	197.6	197.6		201.3	55.9	125.2
15	40.9	40.9		43.1	23.8	26.9
16	24.1	24.4		22.8	28.5	23.1
17	31.6	29.3		22.5	56.0	23.1
18	22.6	29.4		22.4	11.3	32.8
19	14.0	31.8		13.9	39.2	32.8
20		22.7			36.4	56.8
21		14.1			19.2	
22					34.2	
23					26.4	
24					46.0	
25					29.5	
26					19.8	
27					20.2	
28					23.4	
29					11.8	
1′					126.5	
2′					108.7	
3′					143.9	
4′					147.4	
5′					134.0	
6′					102.5	
7′					144.7	
8′					116.2	
9′					166.0	

**Table 5:** Inhibitory effects of the isolates (**1**–**6**) on NO generation by RAW 264.7 murine macrophages in response to lipopolysaccharide (LPS).

Compounds	IC_50_ (μM) ^a^	
	NO	Cell Viability (% Control)
Acrocarpunicain A (**1**)	9.36.± 0.25	100 ± 15.5
Acrocarpunicain B (**2**)	10.11 ± 0.47	95.5 ± 4.2
Acrocarpunicain C (**3**)	5.15 ± 0.18	92.7 ± 4.1
Acrocarpunicain D (**4**)	41.69 ± 3.02	62.2 ± 3.2
Acrocarpunicain E (**5**)	67.38 ± 4.09	86.3 ± 5.4
Acrocarpunicain F (**6**)	27.17 ± 1.87	91.3 ± 7.1
Quercetin ^b^	35.95 ± 2.34	95.9 ± 1.3

^a^ The IC_50_ values were calculated from the slope of the dose-response curves (*SigmaPlot*). Values are expressed as the mean ± S.E.M. of three independent experiments. ^b^ Quercetin was used as a positive control.

## Data Availability

Not applicable.

## Sample Availability

Not available.

## References

[1] Barman D., Dkhar M.S. (2020). Seasonal variation influence endophytic *Actinobacterial* communities of medicinal plants from tropical deciduous forest of Meghalaya and characterization of their plant growth-promoting potentials. Curr. Microbiol..

[2] Singh R., Dubey A.K. (2018). Diversity and applications of endophytic *Actinobacteria* of plants in special and other ecological niches. Front. Microbiol..

[3] Chen C., Ye Y., Wang R., Zhang Y., Wu C., Debnath S.C., Ma Z., Wang J., Wu M. (2018). Streptomyces Nigra Sp. Nov. Is a Novel Actinobacterium Isolated from Mangrove Soil and Exerts a Potent Antitumor Activity in vitro. Front. Microbiol..

[4] Nguyen H.T., Pokhrel A.R., Nguyen C.T., Pham V.T.T., Dhakal D., Lim H.N., Jung H.J., Kim T.S., Yamaguchi T., Sohng J.K. (2020). *Streptomyces* Sp. VN1, a Producer of Diverse Metabolites Including Non-Natural Furan-Type Anticancer Compound. Sci. Rep..

[5] Cheng C., Othman E.M., Stopper H., Edrada-Ebel R.A., Hentschel U., Abdelmohsen U.R. (2017). Isolation of Petrocidin a, a New Cytotoxic Cyclic Dipeptide from the Marine Sponge-Derived Bacterium *Streptomyces* sp. SBT348. Mar. Drugs.

[6] Jinendiran S., Teng W., Dahms H.U., Liu W., Ponnusamy V.K., Chiu C.C.C., Kumar B.S.D., Sivakumar N. (2020). Induction of Mitochondria-Mediated Apoptosis and Suppression of Tumor Growth in Zebrafish Xenograft Model by Cyclic Dipeptides Identified from *Exiguobacterium acetylicum*. Sci. Rep..

[7] Farnaes L., Coufal N.G., Kauffman C.A., Rheingold A.L., Dipasquale A.G., Jensen P.R., Fenical W. (2014). Napyradiomycin Derivatives, Produced by a Marine-Derived Actinomycete, Illustrate Cytotoxicity by Induction of Apoptosis. J. Nat. Prod..

[8] Tamura T., Suzuki S., Hatano K. (2000). Acrocarpospora gen. nov., a new genus of the order Actinomycetales. Int. J. Syst. Evol. Microbiol..

[9] Komaki H., Oguchi A., Tamura T., Hamada M., Ichikawa N. (2021). Diversity of nonribosomal peptide synthetase and polyketide synthase gene clusters in the genus *Acrocarpospora*. J. Gen. Appl. Microbiol..

[10] Meyers P.R. (2015). Analysis of recombinase A (recA/RecA) in the actinobacterial family Streptosporangiaceae and identification of molecular signatures. Syst. Appl. Microbiol..

[11] Niemhom N., Suriyachadkun C., Tamura T., Thawai C. (2013). *Acrocarpospora phusangensis* sp. nov., isolated from a temperate peat swamp forest soil. Int. J. Syst. Evol. Microbiol..

[12] Wayne L.G., Brenner D.J., Colwell R.R., Grimont P.A.D., Kandler O., Krichevsky M.I., Moore L.H., Moore W.E.C., Murray R.G.E., Stackebrandt E. (1987). Report of the Ad Hoc Committee on Reconciliation of Approaches to Bacterial Systematics. Int. J. Syst. Bacteriol..

[13] Balakrishnan B., Lim Y.J., Hwang S.H., Lee D.W., Park S.H., Kwon H.J. (2017). Selective production of red azaphilone pigments in a *Monascus purpureus mppDEG* deletion mutant. J. Appl. Biol. Chem..

[14] Sato K., Goda Y., Sakamoto Sasaki S., Shibata H., Maitani T., Yamada T. (1997). Identification of major pigments containing D-amino acid units in commerical *Monascus* pigments. Chem. Pharm. Bull..

[15] Sweeny J.G., Estrada-Valdes M.C., Iacobucci G.A., Sato H., Sakamura S. (1981). Photoprotection of the red pigments of *Monascus anka* in aqueous media by 1,4,6-trihydroxynaphthalene. J. Agric. Food Chem..

[16] Ohashi M., Kumasaki S., Yamamura S., Nakanishi K., Koike H. (1959). Monascorubrin I: Monascaminone, A Degradation Product. J. Am. Chem. Soc..

[17] Nakanishi K., Ohashi M., Kumasaki S., Yamamura S. (1959). Monascorubrin II: Structures of Monascorubrin and Monascamine. J. Am. Chem. Soc..

[18] Huang Z., Zhang S., Xu Y., Li L., Li Y. (2014). Structural characterization of two new orange pigments with strong yellow fluorescence. Phytochem. Lett..

[19] Cheng M.J., Yang P.H., Wu M.D., Chen I.S., Hsieh M.T., Chen Y.L., Yuan G.F. (2011). Secondary metabolites from the fungus *Monascus purpureus* and evaluation of their cytotoxic activity. Helv. Chim. Acta.

[20] Akihisa T., Yasukawa K., Yamaura M., Ukiya M., Kimura Y., Shimizu N., Arai K. (2000). Triterpene alcohol and sterol ferulates from rice bran and their anti-inflammatory effects. J. Agric. Food. Chem..

[21] Cheng M.J., Cheng Y.C., Hsieh M.T., Chen I.S., Tseng M., Yuan G.F., Chang H.S. (2014). Chemical Constituents of Metabolites Produced by the Actinomycete *Acrocarpospora punica*. Chem. Nat. Compd..

[22] Gieni R.S., Li Y., Hay Glass K.T. (1995). Comparison of [3H]thymidine incorporation with MTT- and MTS-based bioassays for human and murine IL-2 and IL-4 analysis. Tetrazolium assays provide markedly enhanced sensitivity. J. Immunol. Methods.

[23] Johansson M., Kopcke B., Anke H., Sterner O. (2002). Biologically active secondary metabolites from the ascomycete A111-95. 2. Structure elucidation. J. Antibiot..

[24] Berdy J. (2012). Thoughts and facts about antibiotics: Where we are now and where we are heading. J. Antibiot..

[25] Manivasagan P., Venkatesan J., Sivakumar K., Kim S.K. (2014). Pharmaceutically active secondary metabolites of marine *Actinobacteria*. Microbiol. Res..

